# Development and Performance Evaluation of a Gel-Based Plugging System for Complex Fractured Formations Using Acrylic Resin Particles

**DOI:** 10.3390/gels11030162

**Published:** 2025-02-24

**Authors:** Lei Yao, Xiaohu Quan, Jihe Ma, Ge Wang, Qi Feng, Hui Jin, Jun Yang

**Affiliations:** 1 China Oilfield Services Limited (COSL), China National Offshore Oil Corporation, Sanhe 065200, China; 17854227170@163.com (L.Y.); gew35239@gmail.com (J.M.); 2 Oil and Gas Resources Research Center, Ningxia University, Yinchuan 750021, China; 3 Oil & Gas Technology Research Institute, China National Petroleum Corporation Changqing Oilfield Company Limited, Xi’an 710021, China; wangge20220213@163.com; 4 College of Petroleum Engineering, China University of Petroleum (Beijing), Beijing 102249, China; 18563073299@163.com (Q.F.); 2022310126@student.cup.edu.cn (H.J.); 18801281480@163.com (J.Y.)

**Keywords:** acrylic resin, complex fractures, Box–Behnken response, lost circulation control, gel solidification

## Abstract

The issue of fluid loss in fractured formations presents a significant challenge in petroleum engineering, often leading to increased operational costs and construction risks. To address the limitations of traditional lost circulation materials (LCMs) in oil reservoirs with different fracture sizes, this study developed an acrylic resin gel particle with excellent thermal stability (thermal decomposition temperature up to 314 °C) and compatibility. By employing Box–Behnken design and response surface methodology, the synergistic interaction of calcium hydroxide (Ca(OH)_2_), asbestos fibers, and cement was optimized to create a novel gel solidification plugging system that meets the requirements of fluid loss control and compressive strength improvement. Experimental results revealed that the gel-based system demonstrated exceptional performance, achieving rapid fluid loss (total fluid loss time of 18~47 s) and forming a high-strength gelled filter cake (24 h compressive strength up to 17.5 MPa). Under simulated conditions (150 °C), the gel-based system provided efficient fracture sealing, showcasing remarkable adaptability and potential for engineering applications. This study underscores the promise of acrylic resin gel particles in overcoming fluid loss challenges in complex fractured formations.

## 1. Introduction

In the field of petroleum engineering, fractured formations and permeability loss have consistently posed significant technical challenges for operational equipment. These loss-prone formations are typically found in high-pressure and geologically complex environments, such as carbonate formations and structural fracture zones [1,2]. Statistics reveal that more than 40% of fluid loss incidents in global oil and gas fields are associated with these formations, leading to annual unplanned operational costs of billions of dollars [3]. Furthermore, the opening or closing of fracture-related loss channels is highly impacted by formation pressure, with widths varying from tens of microns to several millimeters. Traditional granular bridging materials often lack the size and strength needed to match these gaps, leading to repeated losses [4]. With the progression of oil and gas exploration into deep wells and high-pressure reservoirs, traditional plugging materials face challenges such as low heat resistance and extended curing times, rendering them unsuitable for complex formations. Thus, creating innovative loss control systems designed for high-temperature environments, with both rapid filtration and effective sealing properties, is of great engineering significance [5,6,7].

In recent years, substantial advancements have been achieved in resin-based gel solidification plugging techniques for addressing complex fractured formations [8]. Specifically, acrylic resin, a typical synthetic polymer, has gained broad application in plugging material development for its adjustable molecular structure, outstanding heat resistance, and superior processability [9,10,11]. Zhong et al. [12] developed an oil-absorbing polymer (OAP) that effectively plugs micro-fractures at higher concentrations; its impact on rheology at lower concentrations is minimal, highlighting a need for better dosage control and performance consistency under varied conditions. Bai et al. [13] proposed a water-absorbing resin LCM with excellent absorption and expansion properties. Under high-temperature, high-pressure (HTHP) conditions, its coated shell, which must soften or rupture to function, points to potential vulnerabilities in structural integrity and response predictability. Bai et al. [14] developed a ternary self-expanding oil-absorbing resin to mitigate oil-based drilling fluid losses, optimizing synthesis parameters and exploring its plugging mechanisms. Results revealed excellent oil absorption and thermal stability, yet the specific conditions required for optimal performance indicate limitations in adaptability across different operational environments. Zhu et al. [15] developed an expandable plugging material blending sodium polyacrylate-grafted starch-based resin with rubber, showcasing high-temperature resistance and significant expansion capabilities. However, long-term durability under repetitive stress or varying temperature cycles remains a concern.

Recent studies underscore the necessity of aligning plugging material properties with geological characteristics to optimize performance. Chen et al. [16] explored enhancing temporary plugging agents using a composite of walnut shells and ultrafine CaCO_3_ particles, achieving a plugging rate improvement from 98.10% to 99.81% and withstanding pressures up to 50.39 MPa. This composite also degrades completely at 150 °C within four hours, aligning with technical standards for high-integrity, bidirectional pressure resistance. Hui et al. [17] refined the particle size distribution (PSD) in drilling fluids, demonstrating through orthogonal experiments that optimal PSD significantly mitigates fluid loss, establishing a quantifiable relationship between PSD and fluid retention. Li et al. [18] focused on optimizing particle sizes for carbonate reservoirs, employing response surface methodology to define an optimal PSD that effectively sealed various fracture sizes, achieving a 95.9% acid dissolution rate of the plugging layer, thus enhancing reservoir clean-up and productivity. Nazemi et al. [19] evaluated deformable materials such as rubber and synthetic fibers for their efficacy in fluid loss control within fractured formations. Their findings revealed that a synergistic approach, combining PSD optimization and material selection, offers superior sealing effectiveness and durability over conventional single-material solutions.

Although significant advancements have been made, the development of plugging materials continues to confront challenges, including stability under extreme conditions, improved rapid filtration capabilities, and sustained pressure resistance. This paper tackles these issues by introducing a novel gel-based plugging system incorporating acrylic resin. This system capitalizes on the unique gel-forming properties of the resin and employs formulation optimization to facilitate rapid gelation, ensuring superior adaptability and enhanced pressure resistance. Utilizing response surface methodology (RSM), this study meticulously optimizes the ratios of critical components to refine the gel network structure, offering both theoretical insights and practical applications to overcome plugging challenges in complex loss formations.

## 2. Results and Discussion

### 2.1. Characterization of Acrylic Resin

**Figure 1:**
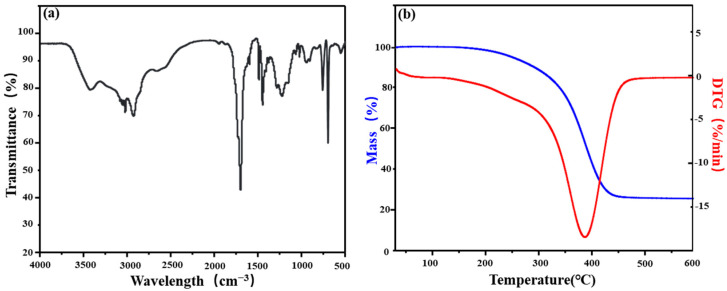
The FTIR (**a**) and TG (**b**) curves of acrylic resin.

As shown in Figure 1a, the sharp peak around 3400 cm^−1^ corresponds to the characteristic peak of hydroxyl (-OH). The region of 2900~3000 cm^−1^ represents the stretching vibrations of C-H bonds found in -CH₃ and -CH₂ groups in the main chain. The peak at 1720 cm^−1^ corresponds to the vibrational absorption of carboxylic acid groups in the monomer. The 1400~1500 cm^−1^ fingerprint region features peaks corresponding to vibrational absorption of hydrogen bonds and carbonyl groups on the benzene ring. The 1000~1300 cm^−1^ region is the stretching vibration zone for ester groups. Analysis indicates that the molecular chain of the acrylic resin contains the functional groups anticipated in the molecular design [20].

If a polymer employed as a plugging agent lacks adequate high-temperature resistance, its performance deteriorates sharply due to molecular structure degradation, reducing the system’s plugging efficiency. Hence, investigating the high-temperature resistance of the acrylic resin particles developed in this work is crucial. Figure 1b illustrates that the thermal degradation of acrylic resin particles occurs in three distinct stages: The first stage, occurring below 314 °C, involves a gradual mass loss (approximately 9.5%), mainly attributed to the evaporation of free and bound water. In the second stage, between 314 °C and 425 °C, the total mass rapidly decreases, with the fastest mass loss rate occurring around 384 °C. The mass loss in this stage reaches 81.7%, attributed to the decomposition of hydroxyl and ester groups with low binding strength in the acrylic resin main chain. The third stage, from 425 to 600 °C, involves further mass loss driven by benzene ring decomposition. Both the main chain and functional side chains break down, resulting in the destruction of the acrylic resin’s core structure and subsequent loss of functionality. The experiments demonstrate that acrylic resin exhibits good thermal stability below 314 °C, proving its significant potential for applications in deep wells and high-temperature environments.

### 2.2. Single-Factor Experimental Analysis

Based on extensive experiments and analyses, asbestos fibers were chosen as the fibrous material to create a network structure, reducing large pores to smaller ones. Acrylic resin was chosen as the granular material for rapid water absorption, expansion, and solidification plugging agents to fill rigid substances in small pores. Cement was employed as the solidification agent to strengthen bonding among fracture walls, fibrous materials, rigid fillers, and soft components. Ca(OH)_2_ was utilized to induce internal self-stress during the cement hardening process, preventing cracks.

**Figure 2:**
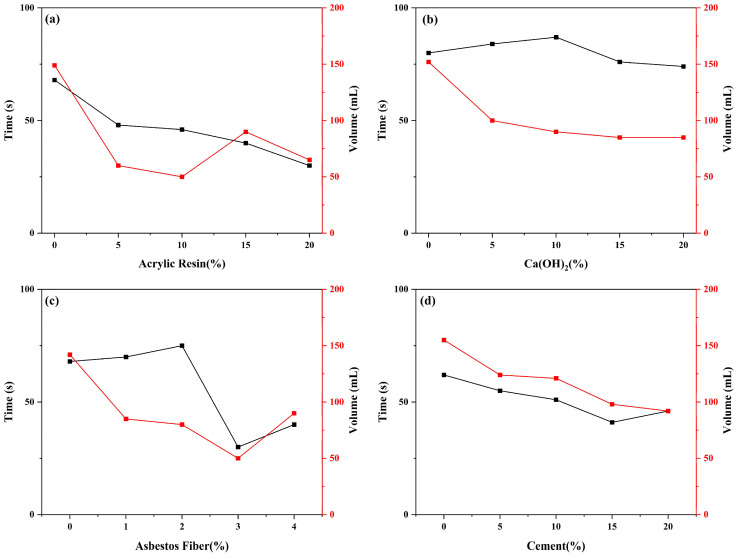
Variation in total filtration time and filtration volume with different ingredients: acrylic resin (**a**), Ca(OH)_2_ (**b**), asbestos fiber (**c**) and cement (**d**).

Single-factor experiments can quickly determine the optimal dosage ranges for acrylic resin, asbestos fibers, cement, and calcium hydroxide. As shown in Figure 2, the overall trend for the total fluid loss time of the four additives is consistent, with the total fluid loss time decreasing as the dosage increases. The rate of fluid loss (total fluid loss time) for the plugging agent is a key determinant of the plugging system’s effectiveness, and it is also a core performance metric in researching rapid fluid loss and solidification systems. Additionally, the total fluid loss volume not only represents the quantity of the plugging system penetrating the loss zone but serves as a vital metric for assessing the plugging layer’s quality. Typically, a denser plugging layer corresponds to reduced fluid loss volume in static conditions [21].

In Figure 2a,c, the filtration volume of acrylic resin and asbestos fibers does not exhibit a monotonous increasing or decreasing trend but instead demonstrates an optimal dosage at which the filtration volume reaches its lowest and most stable value. With the increasing dosage of acrylic resin, the filtration volume decreases at low dosages (0~10%) and stabilizes at 10%, followed by an increase. Similarly, with the addition of asbestos fibers, the filtration volume decreases at low dosages (0~3%), reaching its minimum at 3%, and then increases. The underlying reasons for this phenomenon are as follows: both acrylic resin and asbestos fibers improve the density and strength of the sealing layer through water absorption, filling, and forming a network structure. When added in appropriate amounts, they enhance the sealing efficiency. However, exceeding the optimal concentration likely leads to material aggregation or poor dispersion, thereby impairing the system’s fluidity and uniform distribution. In Figure 2b,d, the total filtration loss and time are shortened with the increase in calcium hydroxide and cement dosage, but the overall change is not large. Based on cost and system liquidity considerations, the maximum dosage cannot be selected as the optimal dosage. Single-factor experimental data indicate that each additive has a different optimal dosage: approximately 10% for acrylic resin, 15% for calcium hydroxide, 3% for asbestos fibers, and 15% for cement. Based on these single-factor results, the sealing performance can be further improved by employing response surface methodology to optimize the synergistic effects of multiple components.

### 2.3. Response Surface Design Results and Error Analysis

Using acrylic resin dosage (A), calcium hydroxide dosage (B), asbestos fiber dosage (C), and cement dosage (D) as independent variables, and total fluid loss time (Y) as the dependent variable, a regression equation was established by fitting the experimental data. The resulting regression equation is expressed as follows:

Fitted model:

Y = 31 − 0.0833A − 0.5B − 1.17C + 1.08D − AB − 0.25AD − 0.25BC + 0.75BD + 0.25

CD + 9.29A^2^ + 7.17B^2^ + 4.67C^2^ + 2.04D^2^

The accuracy of the fitted equation was evaluated by performing error analysis and significance testing on the regression equation coefficients. The variance and significance analysis results of the regression equation are shown in Table 1. Among them, the variance R^2^ is 0.9895; the adjusted variance Adj R^2^ is 0.9791; SS is the sum of squares of variance; DF is the degrees of freedom; MS is the mean square; and Pr > F is the probability of no significant effect.

**Figure 3:**
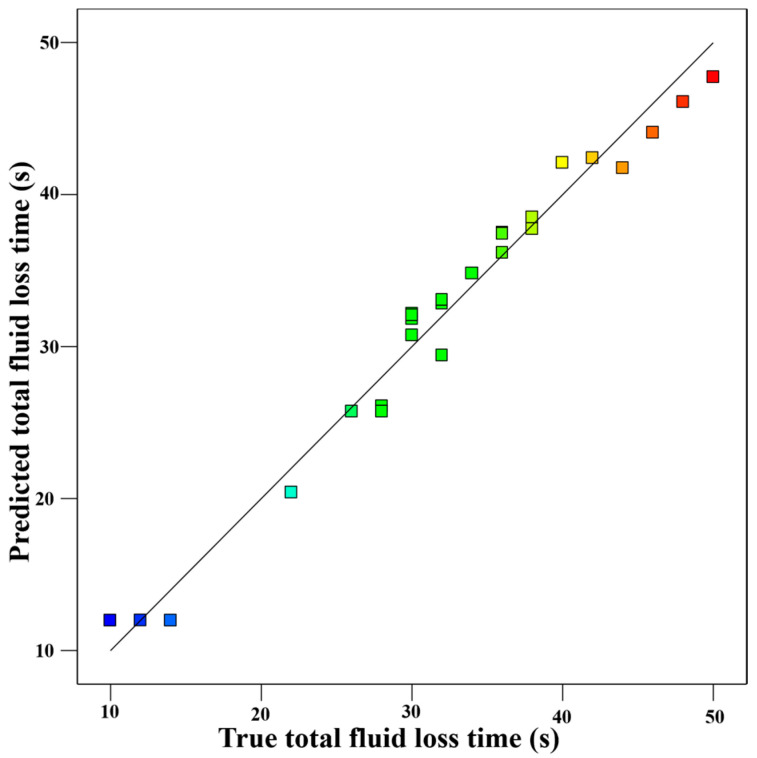
Actual vs. predicted scatter plot.

Figure 3 illustrates the distribution of experimental results and predicted values. The model derived from the experimental data is highly significant, as evidenced by a Pr > F value below 0.05, confirming the significance of the regression coefficients. Moreover, the constructed model exhibits strong adaptability and excellent fitting performance across the entire regression interval. With a variance of R^2^ = 0.973 and adjusted variance Adj R^2^ = 0.9415, the fitted equation demonstrates high accuracy and reliability of the experimental results. The experimentally obtained actual values are evenly distributed and closely align with the predicted values, further confirming the good compatibility between the fitted equation and the experimental results.

### 2.4. Analysis of Interactive Influencing Factors

Following the Box–Behnken design principle, a response surface methodology with four factors and three levels was employed. The total fluid loss time was used as the evaluation criterion. The dosages of acrylic resin, calcium hydroxide, asbestos fibers, and cement were optimized. Additionally, the interactions among the four materials and their effects on total fluid loss time were investigated.

**Figure 4:**
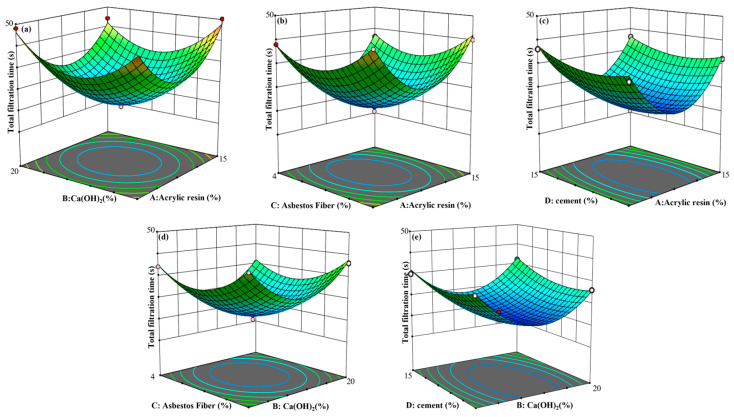
Response surface plots of factor interactions: acrylic resin and Ca(OH)_2_ (**a**), asbestos fiber and acrylic resin (**b**), cement and acrylic resin (**c**), asbestos fiber and Ca(OH)_2_ (**d**), and cement and Ca(OH)_2_ (**e**).

From Figure 4a, when the dosage of asbestos fiber is fixed at 3% and that of cement is fixed at 11.25%, the response surface for acrylic resin is steeper, while that for calcium hydroxide is flatter. This indicates that acrylic resin significantly affects the total fluid loss time, whereas calcium hydroxide has an insignificant effect. Furthermore, the total fluid loss time stays low, with a minimum below 35 s, effectively satisfying the rapid fluid loss criteria. Currently, the synergistic value is −1.0, indicating mutual antagonism between the two. Figure 4b reveals that with calcium hydroxide dosage set at 20% and cement dosage set at 11.25%, the asbestos fiber surface is steeper compared to the flatter surface for acrylic resin. This indicates that asbestos fiber has a greater influence on total fluid loss time than acrylic resin. The synergistic value between the two is 0, indicating that the addition of the two does not affect each other. As illustrated in Figure 4c, with calcium hydroxide dosage at 20% and asbestos fiber dosage at 3%, the acrylic resin surface is steeper compared to the flatter cement surface. This suggests that acrylic resin has a stronger influence on total fluid loss time than cement. Figure 4d shows that with acrylic resin dosage at 10% and cement dosage at 11.25%, the asbestos fiber surface is steeper, whereas the calcium hydroxide surface is flatter. This indicates that asbestos fiber has a greater influence on total fluid loss time than calcium hydroxide. The synergistic values of acrylic resin and cement, asbestos fiber, and calcium hydroxide were all −0.25, indicating that there was a slight antagonistic effect between them, but the numerical value reflected that the effect was not significant. Figure 4e reveals that with the acrylic resin dosage fixed at 10% and asbestos fiber dosage fixed at 3%, the trends of cement and calcium hydroxide dosages on total fluid loss time are comparatively smaller. The promoting effect of calcium hydroxide on cement is obvious and so the synergistic value between the two is 0.75 [22].

From the analysis of the above response surface plots, it is evident that acrylic resin and asbestos fibers are the primary influencing factors. They have a highly significant impact on total fluid loss time. The interaction of these two factors results in the shortest total fluid loss time. This implies that under loss pressure, the plugging slurry can rapidly create a sealing layer. Using the response surface methodology, the independent variables were optimized to the following optimal values: 9.70% acrylic resin, 14.6% calcium hydroxide, 3.13% asbestos fibers, and 10.08% cement. According to field construction recommendations, the finalized optimal parameters were adjusted to 10% acrylic resin, 15% calcium hydroxide, 3% asbestos fibers, and 10% cement.

### 2.5. Evaluation of Loss Circulation Plugging Performance

**Figure 5:**
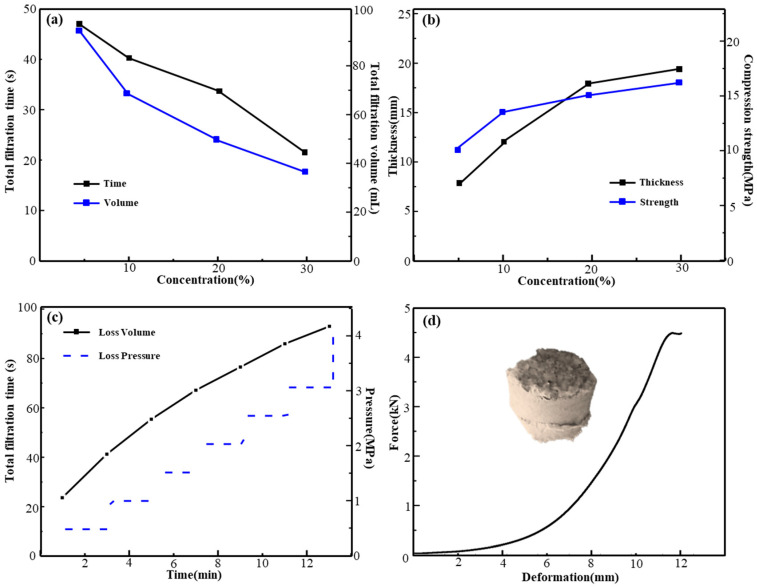
Total loss time and volume (**a**), filter cake thickness and strength (**b**), loss circulation plugging performance under high pressure (**c**), and sand bed filter cake bonding performance (**d**).

As shown in Figure 5a, within the 5~30% concentration range, the total fluid loss time for the rapid fluid loss and solidification plugging slurry is 18~47 s, with a fluid loss volume of 44~96 mL. The short total fluid loss time of the plugging slurry indicates a rapid fluid loss rate, which helps to effectively prevent leakage. With an increase in the plugging agent dosage, the fluid loss volume of the slurry reduces. This reduction is due to the higher solid content in the slurry with increased dosage, forming a denser sealing layer. Therefore, in plugging operations, the dosage of the plugging agent should be adjusted according to specific conditions to achieve optimal plugging performance.

Figure 5b illustrates that as the plugging system dosage rises to 30%, the filter cake’s thickness and compressive strength increase, reaching a maximum compressive strength of 17.5 MPa and a thickness of about 18 mm. This is attributed to the increase in rapid fluid loss and solidification plugging agent, which raises the solid content in the slurry, resulting in a thicker sealing layer and enhanced compressive strength of the filter cake.

The point load system can simulate the fracture process of the plugging slurry filter cake under long-term stress. The strain and stress parameters of the plugging slurry filter cake under compression are measured to assess the compressive strength and stability of the sealing layer. Figure 5c,d show that at a test temperature of 150 °C, the cumulative fluid loss is 96 mL, maintaining pressure for 30 min without breakdown and withstanding over 4 MPa. After 24 h at this temperature, the formed blockage undergoes high-temperature degradation, solidifying and hardening, with compressive strength far exceeding that of the cemented sand bed at room temperature. This is evidently because a dense sealing layer forms on the sand bed under high temperatures. It can effectively block sand beds with particle sizes of 10~20 mesh. Numerous hydrogen bonds on the polymer chains of acrylic resin particles can form a multi-node honeycomb cemented layer structure with the sand bed. This layered cemented structure strengthens the sealing effect of the formation and enhances its stability. This results in a significant improvement in pressure-bearing capacity.

## 3. Conclusions

This research introduces a novel acrylic resin-based gel system tailored for high-temperature and high-pressure (HTHP) environments, incorporating functional groups that enhance their amphiphilicity and thermal resilience. This study utilized response surface methodology to refine the formulation, achieving an optimized balance of components that effectively enhance the plugging capabilities required for challenging geological conditions. The gel system’s rapid formation and robust sealing properties not only meet but exceed the performance parameters typically expected in fractured formations.

The development of this gel-based plugging system represents a significant advancement over traditional materials, providing a durable, efficient solution to the fluid loss challenges prevalent in oilfield operations. The findings from this study not only demonstrate the practical applicability of this innovative gel technology in complex formations but also lay the theoretical groundwork for future advancements in plugging material science. This research paves the way for further exploration into gel-based solutions, potentially transforming practices in the energy sector by improving operational safety and efficiency under extreme conditions.

## 4. Materials and Methods

### 4.1. Materials

The nonionic emulsifier (nonylphenol ethoxylate NP-10), anionic emulsifier (sodium dodecyl diphenyl ether disulfonate), styrene, 2-ethylhexyl acrylate, acrylic acid, and potassium persulfate were all of analytical grade. Ammonia solution, ethanol, and distilled water were common laboratory solvents. Calcium hydroxide (Ca(OH)_2_), asbestos fibers, and cement were industrial-grade products.

### 4.2. Synthesis

A total of 1 g of anionic emulsifier and 2 g of nonionic emulsifier were added to distilled water and stirred until dissolved to form an emulsion. Then, 50 g of styrene, 25 g of 2-ethylhexyl acrylate, and 3 g of acrylic acid were sequentially added. The mixture was mechanically sheared and emulsified, adjusting the pH to 8. After forming the pre-emulsion, it was poured into a reaction flask equipped with a reflux condenser and a stirrer; the reaction system was stirred uniformly at 200 rpm. The temperature was raised to 70 °C, 0.5 g of potassium persulfate was added, and it was stirred continuously for 12 h to synthesize the acrylic resin polymer. The obtained material was filtered using a sieve to collect particles, dispersed with ethanol and distilled water, and centrifuged at 10,000 rpm for 10 min; the precipitate was taken out and vacuum-dried and it was then pulverized to obtain acrylic resin particles.

### 4.3. Characterizations

The acrylic resin particles were dissolved in anhydrous ethanol, followed by multiple washing and precipitation steps to remove impurities and moisture. After drying and pulverizing, the product was pressed into pellets with potassium bromide. The composition and structure were analyzed using a Nicolet Nexus 470 FTIR spectrometer (Thermo Nicolet, Waltham, MA, USA), with a spectral range of 4000~400 cm^−1^. Additionally, to investigate the thermal stability of the prepared acrylic resin particles, thermogravimetric analysis (TGA) was performed using a Shimadzu DTG-60 (Shimadzu Corporation, Kyoto, Japan) under a nitrogen atmosphere. The test conditions included a heating rate of 5 °C/min and a temperature range of 25~600 °C.

### 4.4. Plugging and Filtration Test

The plugging fluid loss experiment was evaluated based on the API fluid loss test method. Quantities of acrylic resin particles, calcium hydroxide (Ca(OH)_2_), asbestos fibers, and cement were individually added to water and stirred until fully dissolved, forming the baseline evaluation system. Subsequently, the impact of varying additive amounts on fluid loss was examined. A pressure of 0.69 MPa was exerted on the baseline evaluation system using an API medium-pressure fluid loss apparatus. The volume of the filtration at different time intervals and the time when filtrate flow stopped (i.e., total fluid loss time, in seconds) were recorded [23].

### 4.5. Response Surface Methodology (RSM) Design

Response surface methodology (RSM) is a predictive modeling approach used to establish complex functional relationships between design variables and response values. Its advantages include fitting simple linear or quadratic polynomials within a localized region, offering convenience in computation and accuracy in prediction. Additionally, it incorporates experimental random errors, improving the precision of the regression model. In contrast to traditional orthogonal experiments, RSM excels in accurately building mathematical models linking design variables and response values. During the process of finding optimal response values and experimental parameters, it allows continuous analysis of design variable levels and the interaction between design factors [24].

This study utilized Design-Expert 13 software for experimental design. The independent variables were the dosages of acrylic resin (A), Ca(OH)_2_ (B), asbestos fibers (C), and cement (D). The experiments were conducted with total fluid loss time as the response variable. The coding and levels of the response surface experimental design factors are shown in Table 2.

### 4.6. Pressure Plugging Experiment

A high-temperature and high-pressure plugging apparatus was used, employing a mixed sand bed (quartz sand mesh ranges of 10–20, 20–40, 40–60, and 80–100) as a simulated loss layer to evaluate the plugging performance of the baseline evaluation system on heterogeneous fractured loss layers. The evaluation process began by blending quartz sand of varying mesh sizes into a sand bed with a total mass of 100 g. This sand bed was then placed at the bottom of the sealed container in the plugging apparatus, and the evaluation system was added to the drilling fluid cup and sealed. Nitrogen gas was connected, and the pressure was adjusted. The release valve was opened to allow nitrogen to enter the drilling fluid cup, and the fluid loss of the system was recorded. Once fluid loss stopped, the pressure was incrementally raised by 1 MPa at 2 min intervals. Fluid loss under different temperature and pressure conditions was documented until the plugging capacity was exhausted or the pressure limit was reached. A cumulative fluid loss curve over time and with different pressure was plotted.

### 4.7. Mechanical Evaluation Experiment

The sand bed filter cake obtained in Section 4.6 was dried and subjected to curing strength evaluation. The JHYL-I dynamic rock strength evaluation system was used to measure the maximum pressure at which the filter cake fractured, referred to as the failure load. The evaluation procedure included opening the compressive strength software, placing the bonded sand bed on the device, and lowering the upper pressure sensor to contact the filter cake surface. Once the instrument was zeroed, the descent rate was set to 1.0 mm/min. The test was initiated and the failure load was recorded when the pressure curve flattened and started to decrease. A curve was plotted showing the 24 h compressive strength of the filter cake as a function of its thickness and the amount of plugging slurry added. The 24 h compressive strength was used as the indicator for evaluating the curing effect.

(1) $$F_{c}=F/A$$

where *F_c_* is the compressive strength (MPa), F is the failure load of the filter cake (kN), and A is the pressure-bearing area of the filter cake (mm^2^).

## Figures and Tables

**Table 1:** ANOVA parameters for regression equation.

Parameter	SS	DF	MS	F	Pr > F
Model	634.05	14	45.29	30.91	<0.0001
A	0.0833	1	0.0833	0.0569	0.8155
B	3.00	1	3.00	2.05	0.1780
C	16.33	1	16.33	11.15	0.0059
D	14.08	1	14.08	9.61	0.0092
AB	4.00	1	4.00	2.73	0.1244
AC	0.0000	1	0.0000	0.0000	1.0000
AD	0.2500	1	0.2500	0.1706	0.6868
BC	0.2500	1	0.2500	0.1706	0.6868
BD	2.25	1	2.25	1.54	0.2390
CD	0.2500	1	0.2500	0.1706	0.6868
A^2^	460.45	1	460.45	314.24	<0.0001
B^2^	273.93	1	273.93	186.94	<0.0001
C^2^	116.15	1	116.15	79.27	<0.0001
D^2^	22.23	1	22.23	15.17	0.0021

**Table 2:** Coded experimental factors and levels.

Factor	Level		
	−1	−1	−1
Acrylic resin (A)	5	10	15
Ca(OH)_2_ (B)	10	15	20
Asbestos fibers (C)	2	3	4
Cement (D)	5	10	15

## Data Availability

Data are contained within the article.

## References

[1] Pang H., Chen M., Wang H., Jin Y., Lu Y., Li J. (2023). Lost circulation pattern in the vug-fractured limestone formation. Energy Rep..

[2] Mirabbasi S.M., Ameri M.J., Alsaba M., Karami M., Zargarbashi A. (2022). The evolution of lost circulation prevention and mitigation based on wellbore strengthening theory: A review on experimental issues. J. Pet. Sci. Eng..

[3] Elkatatny S., Ahmed A., Abughaban M., Patil S. (2020). Deep Illustration for Loss of Circulation While Drilling. Arab. J. Sci. Eng..

[4] Feng Y., Jones J.F., Gray K.E. (2016). A Review on Fracture-Initiation and -Propagation Pressures for Lost Circulation and Wellbore Strengthening. SPE Drill. Complet..

[5] Magzoub M.I., Salehi S., Hussein I.A., Nasser M.S. (2020). Loss circulation in drilling and well construction: The significance of applications of crosslinked polymers in wellbore strengthening: A review. J. Pet. Sci. Eng..

[6] Elahifar B., Hosseini E. (2023). Laboratory study of plugging mechanism and seal integrity in fractured formations using a new blend of lost circulation materials. J. Pet. Explor. Prod. Technol..

[7] Sun J.S., Bai Y.R., Cheng R.C., Lyu K.H., Liu F., Feng J., Lei S.F., Zhang J., Hao H.J. (2021). Research progress and prospect of plugging technologies for fractured formation with severe lost circulation. Pet. Explor. Dev..

[8] Pu L., Xu P., Xu M., Song J., He M. (2022). Lost circulation materials for deep and ultra-deep wells: A review. J. Pet. Sci. Eng..

[9] Yang J., Jiang G., Wang G., Yang L., He Y., Dong T., Yuan X. (2023). Performance evaluation of polymer nanolatex particles as fluid loss control additive in water-based drilling fluids. Geoenergy Sci. Eng..

[10] Sun J.S., Lei S.F., Bai Y.R., Lyu K.H., Cheng R.C., Hao H.J., Liu F. (2023). Research progress and application prospect of functional adhesive materials in the field of oil and gas drilling and production. Pet. Explor. Dev..

[11] Yang J., Bai Y., Sun J., Lv K., Lang Y. (2023). Recent advances of thermosetting resin and its application prospect in oil and gas drilling and production engineering. Geoenergy Sci. Eng..

[12] Zhong H., Shen G., Yang P., Qiu Z., Jin J., Xing X. (2018). Mitigation of Lost Circulation in Oil-Based Drilling Fluids Using Oil Absorbent Polymers. Materials.

[13] Bai X., Deng L., Yan Y., Hu H., Luo Y., Cai X. (2023). Synthesis and performance evaluation of water-absorbing resin coated with polymethyl methacrylate as a lost circulation material. J. Appl. Polym. Sci..

[14] Bai Y., Dai L., Sun J., Lv K., Zhang Q., Shang X., Zhu Y., Liu C. (2022). Experimental study on an oil-absorbing resin used for lost circulation control during drilling. J. Pet. Sci. Eng..

[15] Zhu J., Lou E., Zhang S., Lu H., Wang Z. (2023). Preparation and Performance of Resin-Gel-Rubber Expandable Lost Circulation Material Blend. Gels.

[16] Chen Z., Wu G., Zhou J., Ai C., Zhang A., Xie X., Wu J., Kong X., Li S. (2023). Optimization of degradable temporary plugging material and experimental study on stability of temporary plugging layer. Front. Phys..

[17] Hui C., Ma C., Tian D., Luo C., Zou L., Wang H. An Experimental Study on optimizing the particle size distribution of bridging agents in drilling fluids. Proceedings of the 3rd International Conference on Air Pollution and Environmental Engineering.

[18] Li Z., Zhou Y., Qu L., Wang Y., Zhang S. (2022). A New Method for Designing the Bridging Particle Size Distribution for Fractured Carbonate Reservoirs. SPE J..

[19] Nazemi R., Zargar G., Nooripoor V. (2024). Experimental investigation of deformable additives as loss circulation control agent during drilling and well construction. Sci. Rep..

[20] Yang J., Jiang G.C., Yi J.T., He Y.B., Yang L.L., Dong T.F., Wang G.S. (2024). Natural rubber latex as a potential additive for water-based drilling fluids. Pet. Sci..

[21] Yang J., Dong T., Yi J., Jiang G. (2024). Development of Multiple Crosslinked Polymers and Its Application in Synthetic-Based Drilling Fluids. Gels.

[22] Khormali A., Ahmadi S., Kazemzadeh Y. (2023). Inhibition of Barium Sulfate Precipitation During Water Injection into Oil Reservoirs Using Various Scale Inhibitors. Arab. J. Sci. Eng..

[23] Yang J., Jiang G., Huang S., Yi J., Dong T., He Y., Yang L., Feng Q., Wang G. (2024). Nanobiocatalyst Based on Enzyme Immobilization for Mudcake Removal and Reservoir Damage Control. Energy Fuels.

[24] Antic K., Onjia A., Vasiljevic-Radovic D., Velickovic Z., Tomic S.L. (2021). Removal of Nickel Ions from Aqueous Solutions by 2-Hydroxyethyl Acrylate/Itaconic Acid Hydrogels Optimized with Response Surface Methodology. Gels.

